# Spatio-temporal dynamic of malaria in Ouagadougou, Burkina Faso, 2011–2015

**DOI:** 10.1186/s12936-018-2280-y

**Published:** 2018-04-02

**Authors:** Boukary Ouedraogo, Yasuko Inoue, Alinsa Kambiré, Kankoe Sallah, Sokhna Dieng, Raphael Tine, Toussaint Rouamba, Vincent Herbreteau, Yacouba Sawadogo, Landaogo S. L. W. Ouedraogo, Pascal Yaka, Ernest K. Ouedraogo, Jean-Charles Dufour, Jean Gaudart

**Affiliations:** 1 Aix Marseille Univ, INSERM, IRD, SESSTIM UMR1252 Sciences Economiques & Sociales de la Santé & Traitement de l’Information Médicale, Marseille, France; 2 Embassy of Japan in the Republic of Guinea, Conakry, Guinea; 3 Prospective et Coopération, Laboratoire d’Idées, Bureau d’Etudes Recherche, Marseille, France; 4 Ecole des Hautes Etudes en Santé Publique, Rennes, France; 5 Université libre de Bruxelles, EPS, Centre de Recherche en Epidémiologie, Biostatistique et Recherche Clinique, Brussels, Belgium; 6 IRSS-Clinical Research Unit of Nanoro (IRSS-CRUN), Nanoro, Burkina Faso; 7 IRD, UMR 228 ESPACE-DEV, Station SEAS-OI, Saint-Pierre, France; 8 Programme National de Lutte contre le Paludisme, Ministère de la Santé, Ouagadougou, Burkina Faso; 9 Direction Régionale de la Santé du Centre, Ministère de la Santé, Ouagadougou, Burkina Faso; 10 Direction de la Météorologie, Ministère des Transports, Ouagadougou, Burkina Faso

**Keywords:** Malaria, Spatio-temporal dynamic, Hotspots, Spatial clusters

## Abstract

**Background:**

Given the scarcity of resources in developing countries, malaria treatment requires new strategies that target specific populations, time periods and geographical areas. While the spatial pattern of malaria transmission is known to vary depending on local conditions, its temporal evolution has yet to be evaluated. The aim of this study was to determine the spatio-temporal dynamic of malaria in the central region of Burkina Faso, taking into account meteorological factors.

**Methods:**

Drawing on national databases, 101 health areas were studied from 2011 to 2015, together with weekly meteorological data (temperature, number of rain events, rainfall, humidity, wind speed). Meteorological factors were investigated using a principal component analysis (PCA) to reduce dimensions and avoid collinearities. The Box–Jenkins ARIMA model was used to test the stationarity of the time series. The impact of meteorological factors on malaria incidence was measured with a general additive model. A change-point analysis was performed to detect malaria transmission periods. For each transmission period, malaria incidence was mapped and hotspots were identified using spatial cluster detection.

**Results:**

Malaria incidence never went below 13.7 cases/10,000 person-weeks. The first and second PCA components (constituted by rain/humidity and temperatures, respectively) were correlated with malaria incidence with a lag of 2 weeks. The impact of temperature was significantly non-linear: malaria incidence increased with temperature but declined sharply with high temperature. A significant positive linear trend was found for the entire time period. Three transmission periods were detected: low (16.8–29.9 cases/10,000 person-weeks), high (51.7–84.8 cases/10,000 person-weeks), and intermediate (26.7–32.2 cases/10,000 person-weeks). The location of clusters identified as high risk varied little across transmission periods.

**Conclusion:**

This study highlighted the spatial variability and relative temporal stability of malaria incidence around the capital Ouagadougou, in the central region of Burkina Faso. Despite increasing efforts in fighting the disease, malaria incidence remained high and increased over the period of study. Hotspots, particularly those detected for low transmission periods, should be investigated further to uncover the local environmental and behavioural factors of transmission, and hence to allow for the development of better targeted control strategies.

**Electronic supplementary material:**

The online version of this article (10.1186/s12936-018-2280-y) contains supplementary material, which is available to authorized users.

## Background

In 2015, malaria was the first cause of outpatient consultations (48.0%), hospitalizations (22.6%) and death (23.9%) in Burkina Faso [1]; it was also the main cause of illness among children (80%) [2]. Given the scarcity of resources in the country, malaria treatment requires new strategies that target specific populations, time periods and geographical areas. The World Health Organization (WHO) recommends implementing 2 sets of complementary interventions [3]: (i) universal strategies based on vector control, such as universal distribution of long-lasting insecticide-treated nets (LLINs) and universal access to rapid diagnosis and treatment in health facilities (pillar 1 of the 2015 WHO malaria report); and, (ii) locally tailored strategies that target vulnerable populations (e.g., chemoprevention in pregnant women and children under 5 years), and ones that target spatio-temporal malaria hotspots (with a specific focus on parasites and vectors) (pillars 2 and 3 of the report). For their part, Yukich et al. recommend active case detection and prevalence surveillance at very precise levels of transmission [4]. A number of studies have highlighted the importance of targeting high incidence areas and/or asymptomatic carriers to reduce malaria transmission [5–7]. Others have argued that insofar as spatial heterogeneity gradually increases with the decrease in transmission intensity, intervention programmes should be implemented during low transmission periods [8].

In Burkina Faso, the current national policy is based on universal access to rapid diagnostic test (RDT) and artemisinin-based combination therapy (ACT), and on universal distribution of LLINs [9]. Similar to the situation in other West African countries where malaria transmission is seasonal [10], a strategy targeting children during high transmission periods was implemented in July 2016 in 50 out of 70 health districts (covering a total of 10,874,840 inhabitants) [11]. Each year, this seasonal malaria chemoprevention (SMC) programme covers children aged 3–59 months from July to October [2]. Despite such efforts, the incidence of malaria remains high throughout Burkina Faso. In view of this, malaria treatment requires new, targeted strategies that are based on spatio-temporal assessments of malaria transmission [4].

In Ouagadougou, the capital of Burkina Faso, a previous cross-sectional study (2004) investigating 8 out of 30 neighbourhoods showed that malaria incidence among children (6–12 years) was heterogeneous and associated with lower economic or education levels, distance from hydrological areas, irregularly built-up areas, and lack of LLIN use [12]. However, the dynamic of malaria transmission in the entire central region, including the capital city and the adjacent rural areas has to be explored. In the context of seasonal control strategies, high and low transmission periods need to be properly defined, and the relationships between meteorological factors and onsets of the yearly epidemic need to be better understood, as this will make it possible to anticipate the transmission of the disease.

Lastly, while the spatial pattern of malaria transmission is known to vary depending on local conditions, its temporal evolution has yet to be evaluated. Studies have shown that even at a very local scale, *Anopheles* density and malaria incidence are heterogeneous and associated with spatial and temporal hotspots [8, 13, 14]. Consequently, hotspots should be thoroughly investigated to allow for the development of targeted control strategies [8, 15–17].

The aim of this study was to determine the spatio-temporal dynamic of malaria in the central region of Burkina Faso, taking into account meteorological factors.

## Methods

**Fig. 1:**
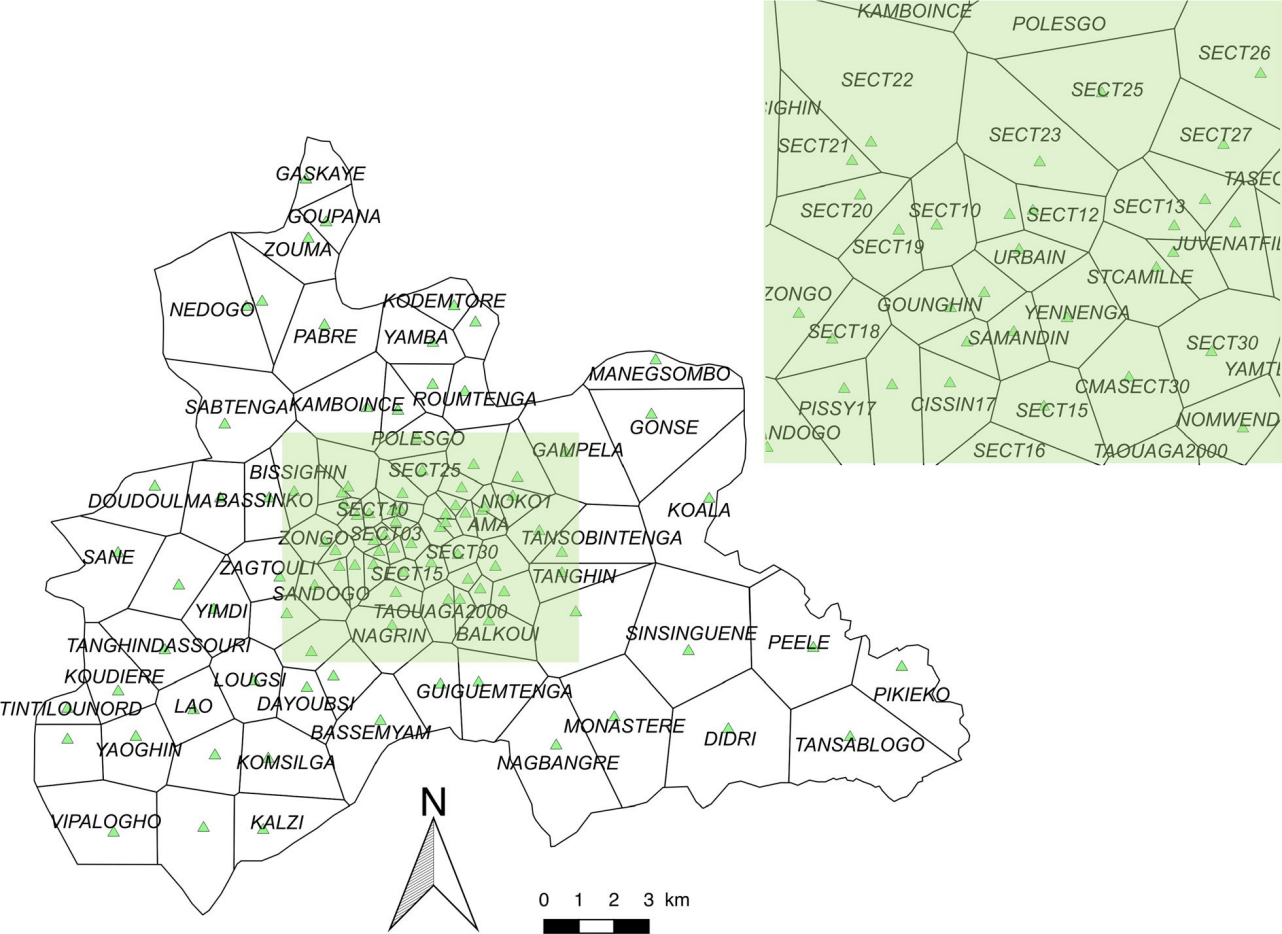
Health area limits and locations of health facilities. Black lines correspond to the limits of the HAs (Thiessen polygons). Each green triangle represents the location of each health facility. The top green rectangle is a zoom of the central urban area (Ouagadougou)

The central region of Burkina Faso has a surface area of 2869 sq km, and includes the capital Ouagadougou (urban area) along with 6 semi-urban or rural provincial departments. In 2015, the population of the region was 2,637,303, representing 14.86% of the national population, and its annual growth rate was 4.2% [1]. The region is divided into 101 health areas (HAs) distributed in 5 health districts (Fig. 1).

The global positioning system (GPS) coordinates of each HA were extracted from the national health map [18] and confirmed by field investigations. Estimated population per HA was extracted from the yearly national action plan for each health district, based on the last census (2006) and the projection (until 2016) released by the *Institut National de la Statistique et de la Démographie* (INSD).

In Burkina Faso, data on malaria incidence can be obtained from 2 sources. The first is the national epidemiological surveillance system, known as the *Télégramme Lettre Officiel Hebdomadaire* (TLOH). In this system, HAs are required to provide weekly reports on 11 diseases (including malaria) to their respective health districts; reported cases are then gathered and controlled by the health districts before being sent to the Ministry of Health. The second is a national database known as ‘BD-Malaria’, which focuses on malaria and is mainly aimed at facilitating the management of RDT and ACT. This database publishes monthly reports on the number of malaria cases, RDT use, treatment stocks (ACT), and the number of LLINs distributed to pregnant women. It relies on the monthly reports provided by all health facilities of Burkina Faso.

In this study, malaria cases were extracted from the TLOH database (weekly) and the BD-Malaria database (monthly) for a 5-year period (2011/1/3–2015/12/27) and for each HA. The 2 databases were compared to validate/correct the weekly number of cases.

Weekly meteorological data were obtained from one meteorological station (*Station de l’Aéroport International de Ouagadougou*) of the *Direction Nationale de la Météorologie* for the same study period. The meteorological variables included were: weekly rainfall (mm), the number of rain events per week, weekly averages of minimum and maximum daily temperature (°C), weekly averages of minimum and maximum daily relative humidity (%), and weekly averages of daily wind speed (km/h).

To begin, meteorological factors were identified using a principal component analysis (PCA) to reduce dimensions and avoid collinearities. The stationarity of the malaria time series and that of the combined meteorological time series derived from the main components were determined with the Box–Jenkins ARIMA modelling procedure (seasonal auto-regressive integrated moving average) [19–21]. The lags between the stationary time series of malaria and the stationary time series of each meteorological factor were measured using cross-correlation functions. Second, the impact of the different meteorological factors on malaria incidence was assessed using a general additive model (GAM). The latter included meteorological components (presenting a significant cross-correlation after the time series was shifted by the time lag), seasonality and trends. A negative binomial distribution was used to account for over-dispersion, and the log-transformed population count was used as an offset to estimate standardized incidence ratios [22]. Furthermore, spline smoothing was performed to capture the non-linear relationship between malaria incidence and combined meteorological factors. Third, a change-point analysis was conducted to detect high, low and intermediate transmission periods (respectively, HTP, LTP and ITP). The change-point analysis in mean and variance was performed using the pruned exact linear time algorithm (PELT) [23].

For each transmission period derived from the change-point analysis, malaria incidence was mapped, and hotspots were identified using Kulldorff’s spatial scan statistic. The latter approach seeks to group the various neighbouring spatial units into potential clusters by moving a scanning window across the geographical region of interest. The algorithm uses circular windows centred at each HA. Potential clusters are defined for a radius ranging from 1 to 50% of the population [24].

The incidences were mapped at the health area scale. Currently, each health facility is associated with an administrative HA defined by the Ministry of Health. The field investigation showed that these administrative boundaries were not relevant as inhabitants mainly accessed the closest health facility, and not the health facility administratively associated to their home. Using the GPS of each health facility, the areas based on the Thiessen polygon approach were estimated. This approach allowed to propose a theoretical area associated with each health facility. Between each point created, corresponding to each health facility, a bisector was drawn to delimit the HA of two adjacent health facilities. Each polygon represented the area around each health facility. The variation in the HA size was explained by the density of health facilities, greater in central urban area than in rural/remote ones.

Spatial cluster analysis was performed using Satscan software version 9.4 (Information Management Services Inc, Silver Spring, Maryland, USA). All other statistical analyses were performed using R v3.3.0 (The R Foundation for Statistical Computing, Vienna, Austria) (packages {mgcv}{caschrono}{FactoMineR}{forecast}). QGIS software (version 2.12.2, Open Source Geospatial Foundation, Boston, USA) was used to provide maps. Figures were formatted with Paint.net software (v4.0.13, Warren Paint & Color Co., Nashville, USA).

## Results

### Overview of the time series

**Fig. 2:**
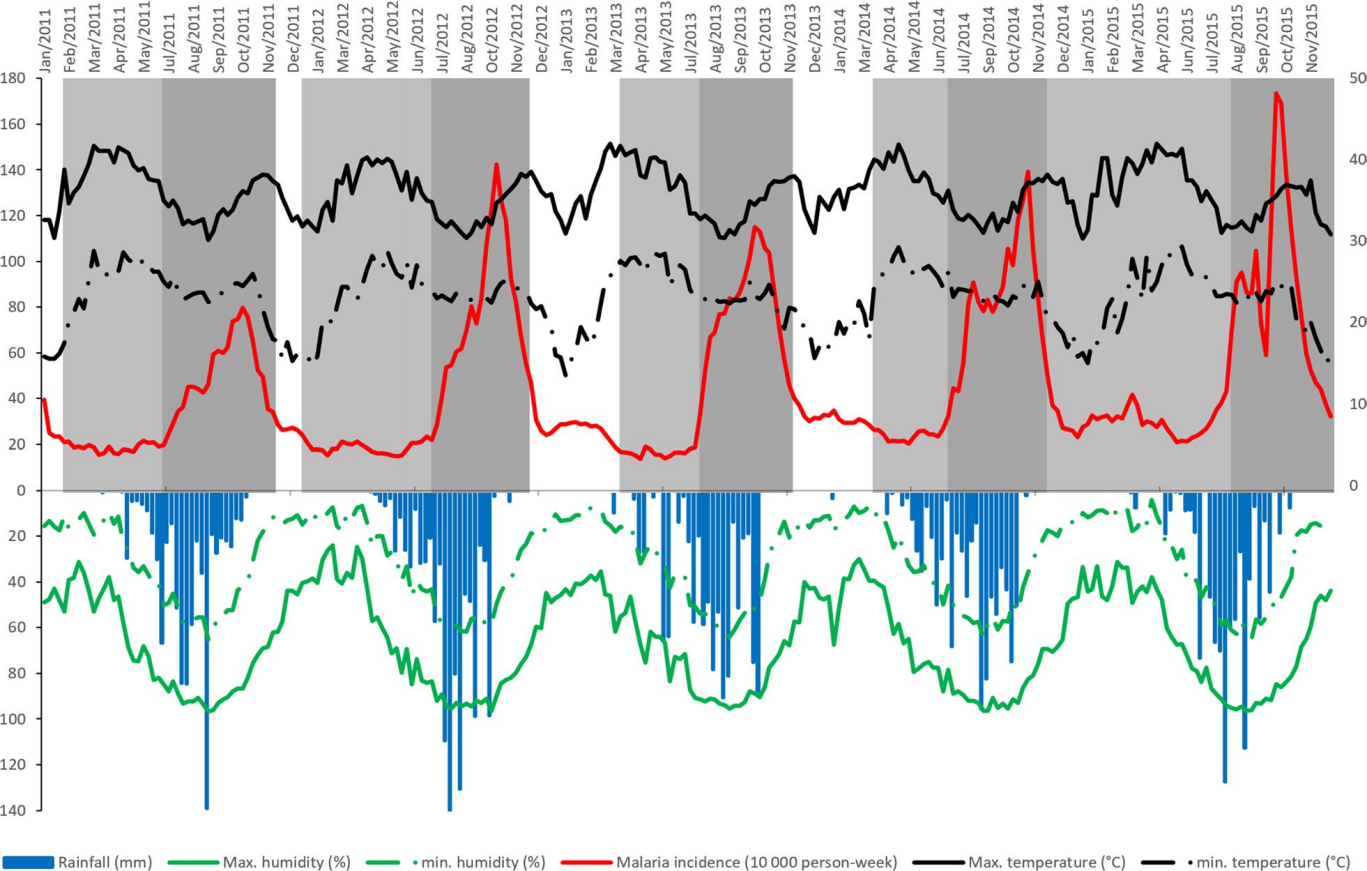
Weekly meteorological factors and malaria incidence from 2011 to 2015. Upper left y-axis represents malaria incidence (1000 person-weeks, red curve); lower left y-axis represents rainfall (mm, blue bar chart), maximum and minimum humidity (%, respectively continuous and dashed green curves); upper right y-axis represents maximum and minimum temperature (°C, respectively continuous and dashed black curves). The white/grey background (upper panel) represents the different transmission periods (white for intermediate, light grey for low, and dark grey for high)

From 2011 to 2015, the malaria incidence time series revealed an association between classical seasonality and dry/rainy annual periodicity (Fig. 2). The highest incidences were observed between June and November. Beyond the classical seasonal pattern, a small rebound was observed just after each annual epidemic. Yet despite the implementation of national control policies during this period, no decreasing trend was observed. Moreover, malaria incidence never went below 13.7 cases/10,000 person-weeks whatever the HA.

### Meteorological data and malaria incidence analysis

Combination of the meteorological factors allowed to identify 3 main components (derived from the PCA, representing 90.17% of the inertia). The first component (53.6% of the inertia) was constituted by rain (rainfall amount and number of rain events) and humidity (maximum and minimum weekly averages). Minimal and maximal temperatures (weekly averages) were combined into the second component (22.16% of the inertia). The weekly average wind speed formed the third component (14.41% of the inertia), see Additional file 1.

After stationarity, the first meteorological component (rainfall, rain events, humidity) was positively and significantly correlated with malaria incidence with a lag of 2 weeks (correlation coefficient: 0.18). The second meteorological component (minimum and maximum temperatures) was negatively and significantly correlated with malaria incidence with a lag of 2 weeks (correlation coefficient: − 0.13). The third component (wind speed) was not significantly correlated with malaria incidence.

**Fig. 3:**
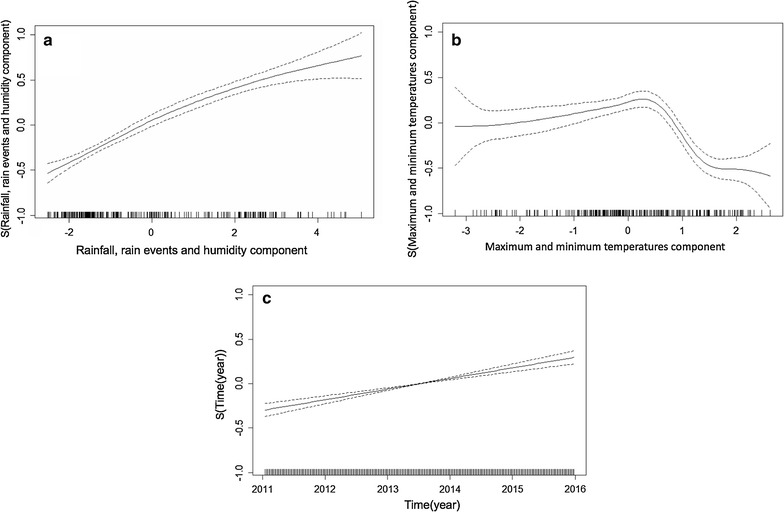
Relationship between malaria incidence and the first meteorological factor (rainfall, rain events, humidity), the second meteorological component (maximum and minimum temperatures), and time. The continuous black curves represent adaptive smooth relationships of malaria incidence according to the first meteorological component (**a**), the second meteorological component (**b**), and time (**c**), with a CI of 95% (dashed black curves)

The multivariate analysis (GAM modelling) assessed the relationship between malaria incidence and the different meteorological components (taking into account the time lag between them), explaining 75% of the deviation. It found a quasi-linear relationship with the first meteorological component (rainfall, rain events, humidity), indicating a significant increase in malaria incidence (p < 0.001; Fig. 3a). The impact of the second meteorological component (minimum and maximum temperatures) was significantly non-linear: malaria incidence increased with temperature but declined sharply with high temperature, indicating a negative impact of high temperature on malaria (p < 0.001; Fig. 3b). A significant positive linear trend (p < 0.001) was also found for the entire time period, indicating an overall increase in malaria incidence in the region (Fig. 3c).

### Spatial hotspot distribution

The change point analysis performed on the malaria incidence time series allowed to identify 3 transmission periods: low, high, and intermediate (Table 1, Fig. 2).

**Table 1:** Malaria incidence and rainfall according to duration, start and end dates for the 3 transmission periods by year

Years	Level of transmission	Duration (weeks)	Start date (day/month/year)	End date (day/month/year)	Malaria incidence per 1000 person-weeks	Rainfall (mm/week)
2011	Intermediate	4	03/01/11	30/01/11	2.86	0
	Low	21	31/01/11	26/06/11	1.89	8.13
	High	22	27/06/11	27/11/11	5.17	29.76
2011–2012	Intermediate	6	28/11/11	08/01/12	2.67	0
	Low	26	09/01/12	08/07/12	1.87	7.92
	High	20	09/07/12	25/11/12	7.88	45.27
2012–2013	Intermediate	16	26/11/12	17/03/13	2.8	0.02
	Low	17	18/03/13	14/07/13	1.68	17.48
	High	20	15/07/13	01/12/13	7.83	34.99
2013–2014	Intermediate	15	02/12/13	16/03/14	3.22	0.23
	Low	15	17/03/14	29/06/14	2.39	13.51
	High	21	30/06/14	23/11/14	8.29	32.52
2014–2015	Low	35	24/11/14	26/07/15	2.99	9.43
	High	22	27/07/15	27/12/15	8.48	25.85

Malaria incidence for LTPs ranged from 16.8 to 29.9 cases/10,000 person-weeks. LTPs generally began in February or March and lasted from 15 to 35 weeks; they overlapped with the dry and hot season for about 3–4 months, until the beginning of the rainy season. Note that the LTP of 2014–2015 was different from the other LTPs, with an observed mean rainfall of 9.43 mm/week.

Malaria incidence for HTPs ranged from 51.7 to 84.8 cases/10,000 person-weeks. HTPs began around the end of June or beginning of July, just after the start of the rainy season, and lasted until the middle or end of November. The duration of HTPs ranged from 20 to 22 weeks, and observed rainfall ranged from 25.85 to 45.27 mm.

ITPs started just after HTPs, and were distinguished from LTPs by significantly higher malaria incidence, which ranged from 26.7 to 32.2 cases/10,000 person-weeks. The duration of ITPs ranged from 4 to 16 weeks. ITPs overlapped with the dry and cold season (December, January, February), for which almost no rain was recorded (i.e., observed mean rainfall ranged from 0 to 0.23 mm/week). Note that no ITP was detected for the year 2014–2015 (see Additional file 2).

### Low transmission periods

**Fig. 4:**
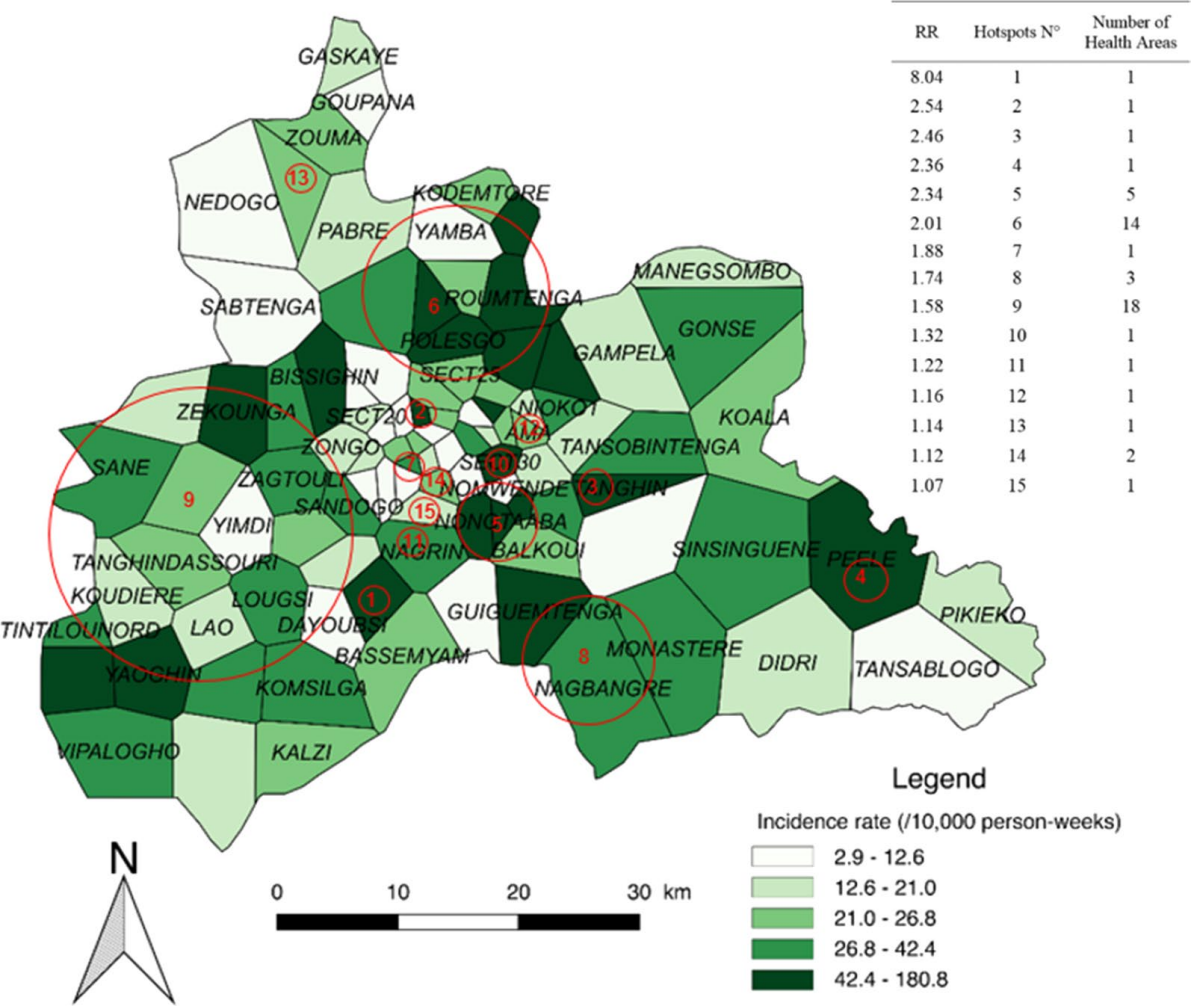
Spatial pattern of incidence per health area and spatial hotspots for low transmission periods. The choropleth map presents the incidence rate (/1000 person-weeks) for the combined LTPs over the 5 years. The red circles represent the high-risk clusters. The attached Table presents the RRs for each hotspot along with the number of HAs

Fifteen significant hotspots including 52 HAs were detected for the combined LTPs, which presented an overall incidence rate of 22.7 cases/10,000 person-weeks. The highest-risk hotspot (Fig. 4, n°1) had a risk ratio (RR) of 8.04 (p < 0.0001) and was composed of one HA (Zeguedesse). This HA was located in a rural area (in the southern and central part of the region), and had an incidence rate of 180.8 cases/10,000 person-weeks for a population of 2969 inhabitants. The largest hotspot (Fig. 4, n°9) was located in a mixed urban/rural area in the western part of the region. It was composed of 18 HAs, and had a RR of 1.58 (p < 0.0001) and an incidence rate of 34.2 cases/10,000 person-weeks.

The rural environment accounted for the highest number of HAs (25); these were located in 6 hotspots presenting an incidence rate of 39.1 cases/10,000 person-weeks (147,013 inhabitants). The urban environment accounted for 20 HAs; these were located in 11 hotspots presenting an incidence rate of 35.3 cases/10,000 person-weeks (557,582 inhabitants) (see Additional file 3).

### High transmission periods

**Fig. 5:**
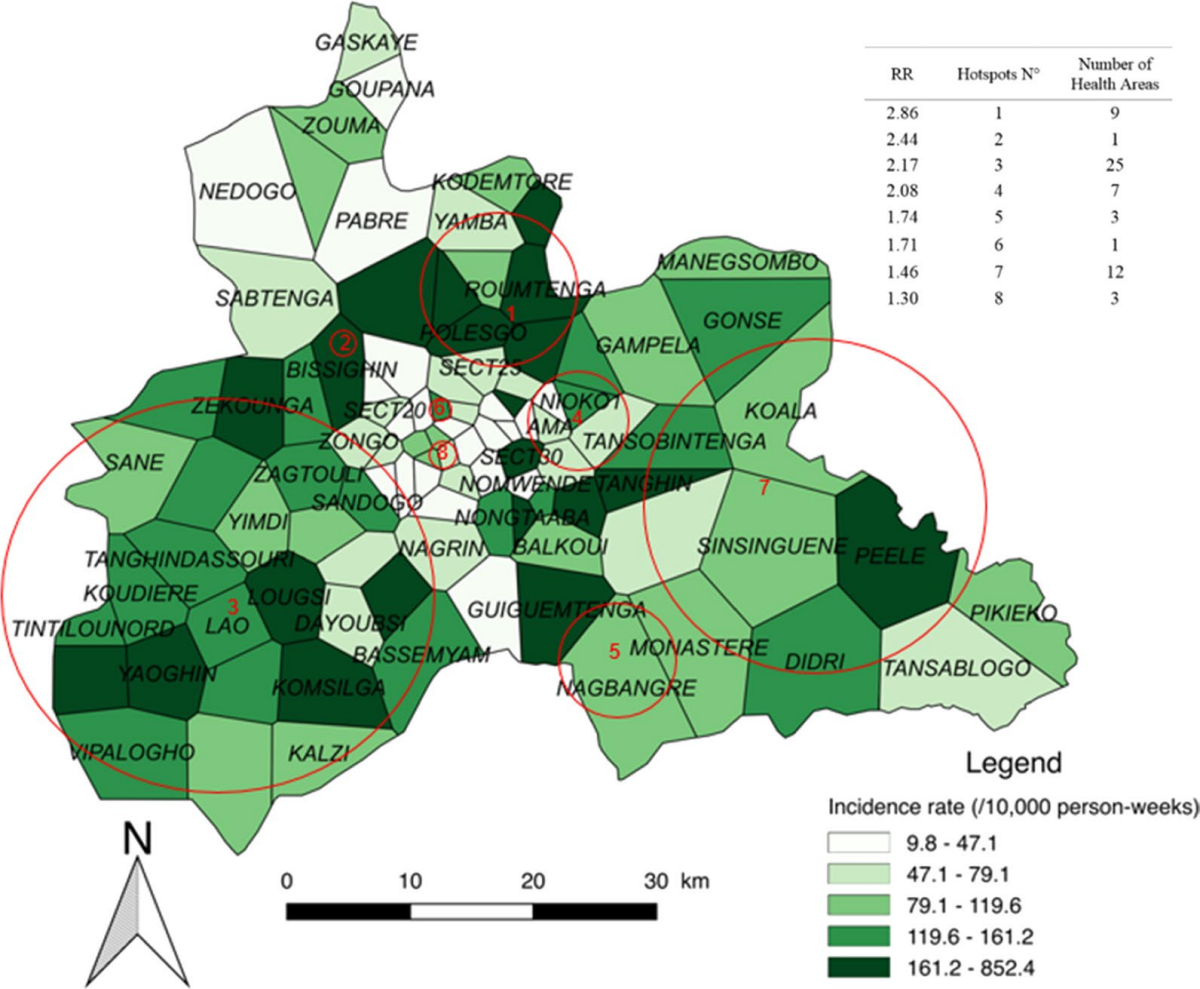
Spatial pattern of incidence per health area and spatial hotspots for high transmission periods. The choropleth map presents the incidence rate (/1000 person-weeks) for the combined HTPs over the 5 years. The red circles represent the high-risk clusters. The attached Table presents the RRs for each hotspot along with the number of HAs

Eight significant hotspots, including 61 HAs, were detected for the combined HTPs, which presented an overall incidence rate of 75.3 cases/10,000 person-weeks. The highest-risk hotspot (Fig. 5, n°1) had a RR of 2.86 (p < 0.0001) and was composed of 9 HAs (see Additional file 3). It was located in the northern part of the region, and had an incidence rate of 206.9 cases/10,000 person-weeks for a population of 53,216 inhabitants. The largest hotspot (Fig. 5, n°3) was located in a mixed urban/rural area in the southwestern and slightly central part of the region. It was composed of 25 HAs, and had a RR of 2.17 (p < 0.0001) and an incidence rate of 146.5 cases/10,000 person-weeks with a population of 224,778 inhabitants.

The rural environment accounted for the highest number HAs (40); these were located in 4 hotspots presenting an incidence rate of 132.8 cases/10,000 person-weeks (381,421 inhabitants). The urban environment accounted for 15 HAs; these were located in 6 hotspots presenting an incidence rate of 141 cases/10,000 person-weeks (413,713 inhabitants) (see Additional file 3). Note that the HA of Zeguedesse, located in hotspot n°3, had the highest incidence rate with 852.4 cases/10,000 person-weeks.

### Intermediate transmission periods

**Fig. 6:**
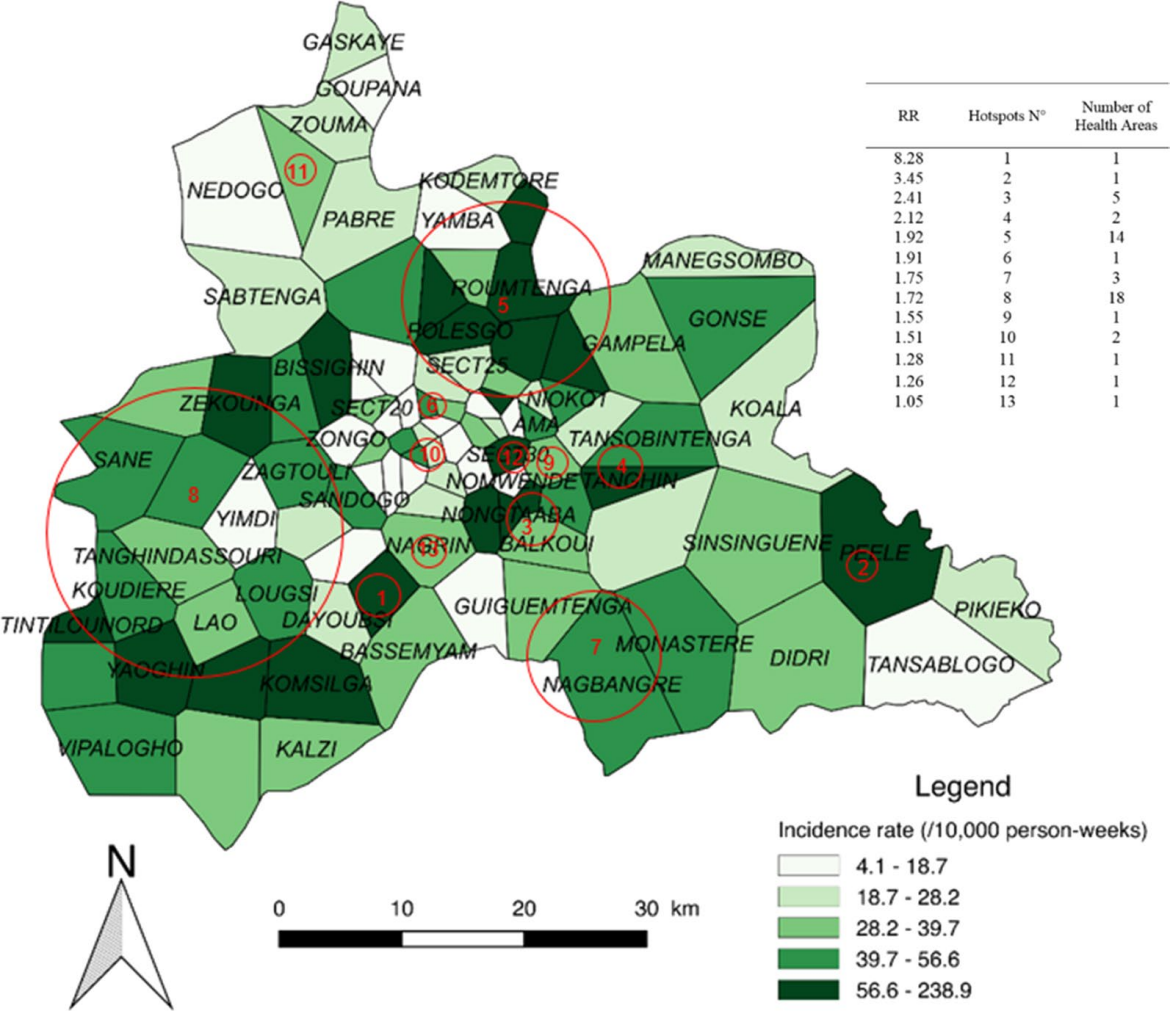
Spatial pattern of incidence per health area and spatial hotspots for intermediate transmission periods. The choropleth map presents the incidence rate (/1000 person-weeks) for the combined ITPs over the 5 years. The red circles represent the high-risk clusters. The attached Table presents the RRs for each hotspot along with the number of HAs

Thirteen significant hotspots including 51 HAs were detected for the combined ITPs, which presented an overall incidence rate of 291 cases/10,000 person-weeks. The highest risk hotspot (Fig. 6, n°1) had a RR of 8.28 (p < 0.0001) and was composed of a single HA (Zeguedesse). This hotspot had an incidence rate of 238.9 cases/10,000 person-weeks for a population of 2969 inhabitants. The largest hotspot (Fig. 6, n°8) was located in a mixed urban/rural area in the western part of the region. It was composed of 18 HAs, and had a RR of 1.72 (p < 0.0001) and an incidence rate of 47.3 cases/10,000 person-weeks (196,730 inhabitants).

The rural environment accounted for the highest number of HAs (27); these were located in 7 hotspots presenting an incidence rate of 55 cases/10,000 person-weeks (38,684 inhabitants). The urban environment accounted for 20 HAs; these were located in 9 hotspots presenting an incidence rate of 48.6 cases/10,000 person-weeks (107,430 inhabitants) (see Additional file 3).

## Discussion

This study highlighted the spatial variability and relative temporal stability of malaria incidence around the capital Ouagadougou, in the central region of Burkina Faso. Despite increasing efforts in fighting the disease, malaria incidence remained high and increased over the study period. The positive quasi-linear relationship between the first meteorological component (rainfall, rain events, humidity) and malaria was similar to that observed elsewhere.

Studies have shown that small puddles of stagnant water exposed to the sun during the rainy season and the beginning of the dry/cold season are favourable to larvae development and mosquito survival in urban settings [25–28]. In this study, no negative impact of strong rains (which can destroy breeding sites) was found [29]. Moreover, the lag of 2 weeks between the first meteorological component (rainfall, rain events, humidity) and malaria incidence was shorter than that reported in other countries assessing rainfall only (a lag of 3 months was reported in Mali [30], and lags of 2–3 months were reported in Ethiopia and East African highlands [28, 31]). This may be due to the presence of permanent water bodies in the region (with 5 dams) and permanent agriculture areas that contribute to a constant presence of vectors at these locations. This may explain the rapid onset of malaria incidence at the beginning of the rainy season. Furthermore, publications assessing the relationship between vegetation and malaria showed similar lags [32–35]. This result could also be due to the analysis at the weekly scale (and not at the monthly scale) and by the use of combinations of meteorological factor (using PCA).

The impact of temperature on malaria incidence has also been highlighted in several studies [36, 37]. In the context of Ethiopia [38], Peterson et al. identified a positive impact of minimum temperature on malaria incidence after a lag of 4 weeks. Entomological studies have stressed the negative impact of high temperature, which increases *Anopheles* death rate [39–41]. Indeed, temperature influences the duration of larvae development, the incubation period of parasites and mosquito survival [27, 37, 42]. Accordingly, the results showed both a positive impact of decreasing temperatures and a negative impact of increasing temperatures on malaria incidence.

While previous studies have found a decreasing impact of wind speed on mosquito survival [43–45], no significant relationship between wind speed and malaria incidence was observed. This results may be due to the higher incidence rate and relatively low wind speeds observed. In fact, during the *harmattan* period (mainly November to March) [46], high wind speed is associated with drought and high temperatures, making it difficult to study the impact of wind speed independently from temperature and drought.

While the impact of meteorological factors on malaria incidence has been the focus of numerous studies, malaria is also caused by other factors, notably parasitaemia and human behaviour. Indeed, humans are the only reservoir of parasites, which means that sub-microscopic and asymptomatic carriage should be investigated for a better understanding of the dynamic of transmission [30, 36, 47]. Nevertheless, meteorological variables can be used to estimate and forecast malaria incidence, thereby providing public health decision-makers with a useful tool [48].

Most published studies (e.g. [12, 49]) describe 2 periods of malaria transmission. By contrast, 3 transmission periods were identified, which did not perfectly correspond to the climatic seasons of Burkina Faso (hot–dry, rainy, cold–dry seasons). HTPs lasted from June to December, whereas the rainy season usually lasts from April to October. This lag should be kept in mind when implementing pre-traveller prevention strategies or programmes of SMC and intermittent treatment and prevention during pregnancy.

The definition of hotspots by using the Satscan method allowed to detect high-risk areas. This method has been developed to detect spatial or space–time clusters of cases, for different distributions [50–52], and for different cluster shapes [53]. This scanning approach makes it possible to overcome the problem of the proximity matrix and the distance weighting function. Based on the likelihood ratio test and a Monte Carlo approach, it allows taking into account the problem of the multiplicity of tests (unlike other scan methods) [24]. But the hotspots definition is relative to the overall incidence and not to particular high-risk places. Even if the method is not constrained by the scanning window shape, the circular or elliptic-shaped scanning window available within the software may impact the results in case of non-circular clusters or edge effects [54]. This approach tends then to detect clusters that are too broad (lack of specificity), by absorbing nearby spatial units [24, 56]. Furthermore, the Satscan performances decrease for low baseline incidences, low sizes of the at-risk population and for low relative risks [55, 57].

Some hotspot locations varied little across the different transmission periods, indicating a relatively stable spatio-temporal pattern. Only the associated relative risks changed across transmission periods, though this was probably due to the method used to estimate these risks.

The specific HA of Zeguedesse (corresponding to hotspot n°1 of the LTPs and to hotspot n°1 of the ITPs) was at higher risk of malaria throughout the 5-year period. This may be partly explained by population growth. Indeed, the construction of a new hospital centre (Centre Hospitalier Universitaire Blaise Compaoré) in 2010 led to the destruction of the villages of Bassemyam and Dayoubsi (114 ha) and to the re-housing of the population in Zeguedesse. The population growth that ensued may have prompted an increase in transmission intensity, and hence in the number of reported cases. However, given that no population census has been conducted since 2010, it may be that the population of Zeguedesse was underestimated, thereby leading to overestimated incidences.

When the transmission periods were compared, 3 zones of particular interest were found. The first zone was located in the southwestern part of the region, and included almost all rural areas of the Boulmiougou health district. Hotspots n°1 and 9 of the LTPs (Fig. 4) were included in hotspot n°3 of the HTPs (Fig. 4), with risk ratios (RRs) increasing from 1.58 and 8.04, respectively, for the LTPs to 2.17 for the HTPs. This zone also corresponded to hotspots n°1 and 8 of the ITPs, which presented RRs of 8.28 and 1.72, respectively. Second, a high-risk area was detected in the northern part of the region, with RRs of 2.01, 2.86 and 1.92 for the LTPs, HTPs, and ITPs, respectively (Figs. 4, 5, 6), corresponding to hotspots n°6, 1, and 5. Third, a stable high-risk area was found in the southeastern part of the region, with RRs of 1.74, 1.74, and 1.75 for the LTPs, HTPs, and ITPs, respectively (Figs. 4, 5, 6), corresponding to hotspots n°8, 5, and 7. In this zone, the location of hotspots n°4 and 2 was the same for the LTPs and the ITPs (Figs. 4, 6).

These 3 zones of interest are located in a similar environment. Indeed, there are 3 dams in the north of the capital, one dam in the southwest (near Boulmiougou), and one dam in the southeast (near Koubri). Furthermore, the eastern part of the region is characterized by the presence of the Nakambe forest. This specific environment may be associated with a higher risk of malaria because it is favourable to the development of *Anopheles* breeding sites [58–61].

## Conclusions

Despite increasing efforts to fight the disease, the incidence of malaria increased between 2011 and 2015 in the central region of Burkina Faso. For each year of the study, 3 periods of malaria transmission were identified: all 3 periods were associated with relatively stable hotspots located in a similar environment (dams). The hotspots detected during the LTPs had a higher incidence of malaria. Future studies should investigate these hotspots to uncover the local environmental and behavioural factors of transmission, as this would allow for the development of better-targeted control strategies. For this purpose, a real-time monitoring system should be implemented based on the existing national monitoring system.

## Additional files

**Additional file 1.** First and second meteorological components derived from the PCA.

**Additional file 2.** Malaria transmission period (low, high, intermediate) by year and season. Malaria incidence (/10,000 person-weeks) is presented for each year and season (dry/hot, rainy, dry/cold) from 2011 to 2015.

**Additional file 3.** Spatial hotspots by transmission period.

## Abbreviations

ACT: artemisinin-based combination therapy
ARIMA: seasonal auto-regressive integrated moving average
GAM: general additive model
GPS: global positioning system
HAs: health areas
HTP: high transmission period
ITP: intermediate transmission period
LLIN: long-lasting insecticide net
LTP: low transmission period
PCA: principal component analysis
PELT: pruned exact linear time
RDT: rapid diagnostic test
RR: risk ratio
SMC: seasonal malaria chemoprevention
TLOH: *Télégramme Lettre Officiel Hebdomadaire*
WHO: World Health Organization
