# Supplementary figures and images for: Spatio-temporal dynamic of malaria in Ouagadougou, Burkina Faso, 2011–2015
Source: Malar J. 2018 Apr 2;17:138. doi: 10.1186/s12936-018-2280-y (PMC5879937; doi:10.1186/s12936-018-2280-y)

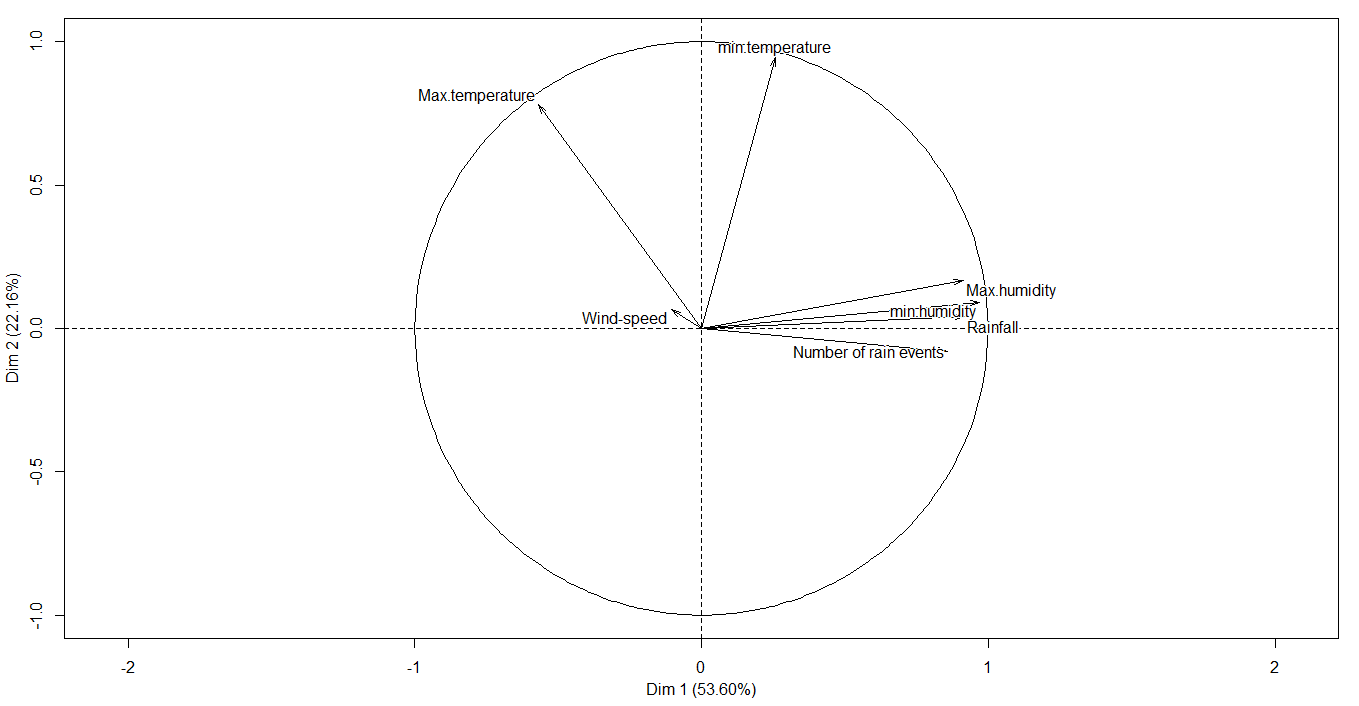

Supplement: Supplementary file 1 — Additional file 1. First and second meteorological components derived from the PCA. [file 12936_2018_2280_MOESM1_ESM.png]
