# Supplementary material for: Spatio-temporal dynamic of malaria in Ouagadougou, Burkina Faso, 2011–2015
Source: Malar J. 2018 Apr 2;17:138. doi: 10.1186/s12936-018-2280-y (PMC5879937; doi:10.1186/s12936-018-2280-y)
Supplement: Supplementary file 2 — Additional file 2. Malaria transmission period (low, high, intermediate) by year and season. Malaria incidence (/10,000 person-weeks) is presented for each year and season (dry/hot, rainy, dry/cold) from 2011 to 2015. [file 12936_2018_2280_MOESM2_ESM.docx]

Additional file 2: Malaria transmission period (Low, Intermediate and High Transmission Periods) by years and seasons. Malaria incidences (/10,000 person-weeks) are presented for each year and season (dry/hot, rainy, dry/cold) from 2011 to 2015.

| Seasons | | dry / cold | dry / hot | | | | rainy | | | | | dry / cold | |
| --- | --- | --- | --- | --- | --- | --- | --- | --- | --- | --- | --- | --- | --- |
| Months | | 1 | 2 | 3 | 4 | 5 | 6 | 7 | 8 | 9 | 10 | 11 | 12 |
| Year | 2011 | ITP (28.6) | LTP (18.9) | | | | | HTP (51.7) | | | | | ITP… |
|  | 2012 | …ITP (26.7) | LTP (18.7) | | | | | | HTP (78.8) | | | | ITP… |
|  | 2013 | …ITP (28.1) | | | LTP (16.8) | | | | HTP (78.3) | | | | ITP… |
|  | 2014 | …ITP (32.2) | | | LTP (23.9) | | | HTP (82.9) … | | | | | LTP… |
|  | 2015 | …LTP (29.9) | | | | | | | HTP (84.8) | | | | |
