# Supplementary material for: Spatio-temporal dynamic of malaria in Ouagadougou, Burkina Faso, 2011–2015
Source: Malar J. 2018 Apr 2;17:138. doi: 10.1186/s12936-018-2280-y (PMC5879937; doi:10.1186/s12936-018-2280-y)
Supplement: Supplementary file 3 — Additional file 3. Spatial hotspots by transmission period. [file 12936_2018_2280_MOESM3_ESM.docx]

Additional file 3: Spatial hotspots by Transmission periods

| Low Transmission Period | | | |
| --- | --- | --- | --- |
| Risk Ratios (RR) | Hotspot number | Health area (HA) name | Urbanisation level ( number of HA) |
| 8.04 | 1 | Zeguedesse | Rural (1) |
| 2.54 | 2 | Pogbi | Urban (1) |
| 2.46 | 3 | Tanghin | Urban (1) |
| 2.36 | 4 | Peele | Rural (1) |
| 2.34 | 5 | Nomwende, Tasect30, Nongtaaba, Sect30, Taouaga2000 | Urban (5) |
| 2.01 | 6 | Roumtenga, Songdin, Yamba, Polesgo, Sakoula, Sagnioniogo, Kodemtore Kamboince, Wendefande, Dassasgho, Nioko1,Sect26, , Sect25, Sect27 | Rural (7)  Semi-urban (4)  Urban (3) |
| 1.88 | 7 | Sect08 | Urban (1) |
| 1.74 | 8 | Nagbangre, Monastere, Guiguemtenga | Rural (3) |
| 1.58 | 9 | Sane, Bazoule, Doudoulma, , Yimdi, Zekounga, Koudiere, Tintilounord ,Lao, Boassa, Lougsi, Yaoghin, Tintilousud, Bassinko, Tanghindassouri, Bissighin, Zagtouli, Sabtenga, Sandogo | Rural (12),  Semi-urban (3),  Urbain (3) |
| 1.32 | 10 | Stcamille | Urban (1) |
| 1.22 | 11 | Nagrin | Urban (1) |
| 1.16 | 12 | AMA | Urban (1) |
| 1.14 | 13 | Zibako | Rural (1) |
| 1.12 | 14 | Sect03, Gounghin | Urban (2) |
| 1.07 | 15 | Sect15 | Urban (1) |
| High Transmission Period | | | |
| Risk Ratios (RR) | Hotspot number | Health area (HA) name | Urbanisation level ( number of HA) |
| 2.86 | 1 | Sagnioniogo, Kodemtore, Yamba, Roumtenga, Songdin, Sakoula, Polesgo, Kamboince, Sect26 | Rural (7),  Semi-urban (1),  Urban (1) |
| 2.44 | 2 | Dassasgho | Urban (1) |
| 2.17 | 3 | Tintilousud, Tintilounord, Yaoghin, Koudiere, Vipalogho, Lao, Tampoussoumdi, Tampouy, Bazoule, Lougsi, Sane, Yimdi, Komsilga, Kalzi, Dayoubsi, Boassa, Tiguindalgue, Doudoulma, Zeguedesse, Zekounga, Bassemyam, Tanghindassouri, Bassinko ,Zagtouli, Sandogo | Rural (21),  Semi-urban (2),  Urban (2) |
| 2.08 | 4 | Nongtaaba, Taouaga2000, Nomwende, Tasect30, Balkoui, Dagnongo, Sect30 | Urban (7) |
| 1.74 | 5 | Nagbangre, Monastere, Guiguemtenga | Rural (3) |
| 1.71 | 6 | Pogbi | Urban (1) |
| 1.46 | 7 | Koala, Gonse, Sinsinguene, Gampela, Peele, Tansobintenga, Tanghin, Tanlarghin, Saaba, Didri, Nioko1, Wendefande | Rural (10),  Semi-urban (2) |
| 1.3 | 8 | Gounghin, Sect03, Sect08 | Urban (3) |
| Intermediate Transmission Period | | | |
| Risk Ratios (RR) | Hotspot number | Health area (HA) name | Urbanisation level ( number of HA) |
| 8.28 | 1 | Zeguedesse | Rural (1) |
| 3.45 | 2 | Peele | Rural (1) |
| 2.41 | 3 | Nomwende, Tasect30, Nongtaaba, Sect30, Taouaga2000 | Urban (5) |
| 2.12 | 4 | Tanghin, Tansobintenga | Urban (1),  Rural(1) |
| 1.92 | 5 | Roumtenga, Songdin, Yamba, Polesgo, Sakoula, Sagnioniogo, Kodemtore, Kamboince, Wendefande, Nioko1, Sect26, Sect25, Sect27, Dassasgho, | Rural (7),  Semi-urban (3),  Urban (4) |
| 1.91 | 6 | Pogbi | Urban (1) |
| 1.75 | 7 | Nagbangre, Monastere, Guiguemtenga | Rural (3) |
| 1.72 | 8 | Sane, Bazoule, Doudoulma, Tanghindassouri, Yimdi, Zekounga, Koudiere, Tintilounord, Lao, Boassa, Lougsi, Yaoghin, Tintilousud, Bissighin, Bassinko, Zagtouli, Sabtenga, , Sandogo | Rural (13),  Semi-urban (1),  Urbain(4) |
| 1.55 | 9 | AMA | Urban (1) |
| 1.51 | 10 | Sect08, Gounghin | Urban (2) |
| 1.28 | 11 | Zibako | Rural (1) |
| 1.26 | 12 | Stcamille | Urban (1) |
| 1.05 | 13 | Nagrin | Urban (1) |
